# Δ^1^-Pyrroline-5-Carboxylate/Glutamate Biogenesis Is Required for Fungal Virulence and Sporulation

**DOI:** 10.1371/journal.pone.0073483

**Published:** 2013-09-09

**Authors:** Ziting Yao, Chengwu Zou, Hui Zhou, Jinzi Wang, Lidan Lu, Yang Li, Baoshan Chen

**Affiliations:** State Key Laboratory for Conservation and Utilization of Subtropical Agro-Bioresources, Key Laboratory of Ministry of Education for Microbial and Plant Genetic Engineering, College of Life Science and Technology, Guangxi University, Nanning, China; Soonchunhyang University, Republic of Korea

## Abstract

Proline dehydrogenase (Prodh) and Δ^1^-pyrroline-5-carboxylate dehydrogenase (P5Cdh) are two key enzymes in the cellular biogenesis of glutamate. Recombinant Prodh and P5Cdh proteins of the chestnut blight fungus *Cryphonectria parasitica* were investigated and showed activity in *in vitro* assays. Additionally, the *C. parasitica Prodh* and *P5Cdh* genes were able to complement the *Saccharomyces cerevisiae put1* and *put2* null mutants, respectively, to allow these proline auxotrophic yeast mutants to grow on media with proline as the sole source of nitrogen. Deletion of the *Prodh* gene in *C. parasitica* resulted in hypovirulence and a lower level of sporulation, whereas deletion of *P5Cdh* resulted in hypovirulence though no effect on sporulation; both Δ*prodh* and Δ*p5cdh* mutants were unable to grow on minimal medium with proline as the sole nitrogen source. In a wild-type strain, the intracellular level of proline and the activity of Prodh and P5Cdh increased after supplementation of exogenous proline, though the intracellular Δ^1^-pyrroline-5-carboxylate (P5C) content remained unchanged. *Prodh* and *P5Cdh* were both transcriptionally down-regulated in cells infected with hypovirus. The disruption of other genes with products involved in the conversion of arginine to ornithine, ornithine and glutamate to P5C, and P5C to proline in the cytosol did not appear to affect virulence; however, asexual sporulation was reduced in the Δ*pro1* and Δ*pro2* mutants. Taken together, our results showed that *Prodh, P5Cdh* and related mitochondrial functions are essential for virulence and that proline/glutamate pathway components may represent down-stream targets of hypovirus regulation in *C. parasitica*.

## Introduction

Proline dehydrogenase (Prodh), also known as proline oxidase (POX) in mammals, was first isolated in 1978 from *Escherichia coli* and shown to have the ability to catalyze the conversion of proline to Δ^1^-pyrroline-5-carboxylate (P5C) [1]. A year later, the ortholog of Prodh in the yeast *Saccharomyces cerevisiae, Put1*-encoded proline dehydrogenase, was shown to be able to convert proline to P5C, which is then converted to glutamate by P5C dehydrogenase (P5Cdh) encoded by *Put2* [2]. The *Put1* gene is present as a single copy in the yeast genome [3], and its product possesses a membrane anchor that causes it to loosely associate with the inner mitochondrial membrane [4]. Intracellular L-proline was found to accumulate to a higher level in *put1* mutants, conferring increased tolerance to freezing and desiccation stress [5] and inhibition to cellular apoptosis induced by H_2_O_2_ [6]. Put2 localizes to the mitochondrial matrix and was shown to enhance cell growth by improving anaerobic arginine catabolism [7]. Under aerobic conditions, *put2* mutants suffer from cellular toxicity due to the accumulation of P5C, which induces an increased level of reactive oxygen species (ROS) in the cell [8]. However, the biological functions of Prodh and P5Cdh have not been previrously reported in the pathogenic fungi.

*Cryphonectria parasitica* is the pathogen responsible for the destructive chestnut blight that swept the once-dominant chestnut forests in North America, and interactions between *C. parasitica* and its host have been a major focus of modern plant pathology since its first report in 1904 [9]. Efforts in genetics [10], [11], biochemistry [12], molecular biology [13], and, recently, “omics” [14], [15] have been put forward to elucidate the regulation of *C. parasitica* virulence with regard to chestnut. Of particular significance is the discovery of hypoviruses and their use in the dissection of the components and regulation of fungal virulence [16]. It is now known that the trimeric G protein signaling pathway [17]–[21], the inositol triphosphate (IP_3_)/Ca^2+^/calmodulin signaling pathway [22], and the mitogen-activated protein kinase (MAPK) signaling pathway are essential for *C. parasitica* virulence [23]–[26]. In addition to these pathways, genes functioning in the methylation pathway [27] and in apoptosis [28] were also found to be required for virulence.

A mitochondrial dysfunction mutant [29] was shown to have a hypovirulence phenotype and gene expression patterns very similar to those caused by hypovirus infection [30], suggesting that a hypovirus may exert its effect by perturbating an important mitochondrial function. However, this mitochondrial function that is essential for *C. parasitica* virulence remains unknown. Additionally, the association of Prodh and P5Cdh with hypovirus has not yet been reported.

In this study, we analyzed the effects of the genes involved in the proline/glutamate pathway on virulence and other traits and on global gene expression patterns in *C. parasitica* using gene knockout technology. We demonstrate that *Prodh* and *P5Cdh* are essential for virulence and that *Prodh, Pro1* and *Pro2* are required for sporulation in *C. parasitica*. Evidence was also obtained showing that the accumulation of both *Prodh* and *P5Cdh* transcripts was suppressed by hypovirus infection.

## Materials and Methods

### Fungal strains and growth conditions

The hypovirus CHV1-EP713-infected *C. parasitica* strain EP713 (ATCC 52571), its isogenic virus-free parent EP155 (ATCC 38755), and a highly efficient homologous recombination strain, Δ*ku80* [31], were maintained on PDA (Difco) plates under a constant light-dark (12 h/12 h) cycle at 25°C. For liquid culture, EP complete medium [32] was used, and the cultures were incubated at 28°C with shaking at 200 rpm. Preparation of the primary inocula for liquid cultures was performed as previously described [33]. Radial growth on the plates was assessed by measuring the diameter of the colonies [34].

### Alignment and phylogenetic analysis

Prodh and P5Cdh in the species of interest were identified by searching the NCBI genomic BLAST databases (http://blast.ncbi.nlm.nih.gov/Blast.cgi), and the amino acid (aa) sequences of the proteins of interest were downloaded from the NCBI protein database (http://www.ncbi.nlm.nih.gov/protein/). Alignment was performed using the alignment program in the Vector NTI 11.0 software. Phylogenetic trees were constructed with amino acid sequences using Neighbor-joining, minimum-evolution, and maximum-parsimony methods in the MEGA4.0 software. The sequence alignment data were bootstrapped with 1,000 resamplings of the alignments to assess the robustness of the lineages in the trees. The trees were visualized using the software TreeView.

### Prodh and P5Cdh enzymatic activity assays

Total RNA was isolated from vegetative hyphae as previously described [33], and cDNA was prepared using the First Strand cDNA Synthesis Kit from Roche Applied Science (Mannheim, Germany). The cDNA of Prodh (JGI protein ID 277618) from *C. parasitica* without the mitochondrial transit peptide (mTP) (aa positions 1–21) and the cDNA of P5Cdh (JGI protein ID 344979) from *C. parasitica* without the mTP (aa positions 1–45) were generated by site-directed mutagenesis PCR; the resulting fragments were named ProdhΔ21 and P5CdhΔ45, respectively. ProdhΔ21 and P5CdhΔ45 were subcloned into the pGEX-4T-1 vector using the *Eco*R I, *Hind* III, and *Sal* I restriction sites to generate recombinant expression constructs pGEX-4T-1- ProdhΔ21 and pGEX-4T-1- P5CdhΔ45, respectively. Positive clones were selected by transformation of the constructs into *E. coli* BL21 (DE3) pLysS and screened on LA plates containing chloramphenicol (34 μg/mL) and ampicillin (50 μg/mL). The selected single colonies were inoculated into 5 mL of LB broth containing chloramphenicol and ampicillin at appropriate concentrations and incubated overnight to generate the primary inocula. A primary inoculum of 10 mL was then used to inoculate 1 L of LB containing the necessary antibiotics and incubated at 37°C with shaking at 200 rpm until OD_600_ = 0.4; ProdhΔ21 expression was then induced with 0.1 mM IPTG for 6 h at 16°C and P5CdhΔ45 expression with 0.3 mM IPTG for 8 h at 18°C. The bacteria were then collected by centrifugation at 2,500×g for 10 min at 4°C. A 1-g bacterial pellet was resuspended in 10 mL of PBS buffer, and lysozyme and DNase I were added to a final concentration of 0.2 mg/mL and 20 μg/mL, respectively. The cell suspension was gently stirred for 30 min at 4°C and then centrifuged at 12, 000 × g for 10 min at 4°C to remove the cell debris. The supernatant was applied to a GST Sefinose resin column from Sangon Biotech (Shanghai, China) for affinity purification of the recombinant protein. The recombinant proteins were eluted with 10 mM reduced glutathione in 50 mM Tris-HCl (pH 8.0). The quality of recombinant proteins was analyzed by SDS-PAGE. The ProdhΔ21 protein was further concentrated using an Amicon 10-kDa cutoff filter from Millipore Corporation (Massachusetts, USA), and the P5CdhΔ45 protein was concentrated using an Amicon 30-kDa cutoff filter. The enzymatic activity of 2 μM ProdhΔ21 protein was assayed in 5 mM Tris-HCl (pH 8.0), 8 mM MgCl_2_, 2 mM DTT, 20 μM FAD, and 4 mM 2-aminobenzaldehyde (2-AB); the *K*_m_ of ProdhΔ21 was determined at 30°C using proline as a substrate. The enzymatic activity of 2.2 μM P5CdhΔ45 protein was assayed in 5 mM HEPES (pH 7.5), 1 mM MgCl_2_, 0.5 mM DTT, and 0.4 mM P5C (freshly prepared and adjusted to pH 7.0 with 10 M KOH before use); the *K*_m_ of P5CdhΔ45 was measured at 30°C using NAD as a substrate. The absorbance of P5C-2-AB was measured at 443 nm (ε_443_ = 2590 cm^–1^·M^–1^), and the absorbance of NADH was measured at 340 nm (ε_340_ = 6220 cm^–1^·M^–1^) using a BIO-TEK μQuant microplate reader; the data were analyzed using KC junior software.

The mycelial activity of Prodh and P5Cdh was assayed according to an established protocol used in yeast [2]. Briefly, a 7-day-old mycelium grown on a PDA plate was collected, and 0.2 g of mycelium was immediately immersed in liquid nitrogen for 10s and then quickly ground into a powder. The powder was placed into an EP tube containing 1 mL of 0.1 M HEPES buffer (pH 7.5) with 3 mM MgCl_2_ and kept on ice. The samples were vortexed vigorously to break the cell wall; the mixture was centrifuged at 8000×g to remove cell debris, and the supernatant was used in enzymatic assays. For the Prodh activity assay, 0.4 mL of 10% proline was added to 0.4 mL of supernatant, vortexed for 10 s, and incubated without shaking at 30°C for 30 min. The assay was developed colorimetrically by the addition of 0.1 mL of 2-AB (6 mg/mL in 20% ethanol) to the reaction mixture; the reaction proceeded for 30 min before being terminated by the addition of 0.5 mL of 10% trichloroacetic acid. The absorbance of the supernatant was measured at 443 nm (ε_443_ = 2590 cm^–1^·M^–1^). The P5Cdh activity was measured by determining the P5C-dependent reduction of NAD. A 4-μL aliquot of 50 mM P5C and 30 μL of 50 mM NAD were added to 0.4 mL of cell extract, mixed by vortexing vigorously for 10 s, and incubated without shaking at 30°C for 30 min. The absorbance of the supernatant was measured at 340 nm (ε_340_ = 6220 cm^–1^·M^–1^).

### Complementation of yeast mutants

The *S. cerevisiae* strains 24099 and 21000 (His^–^, Leu^–^, Ura^–^) and their parent strain BY4743 were purchased from Invitrogen Corporation (California, USA). The sequences of *Put1, Put2* and *Sdh1*-mTP (1–156bp of the coding sequence) from yeast were amplified using genomic DNA, and *Prodh* and *P5Cdh* were amplified using *C. parasitica* mRNA with the primers listed in Table S1. The full-length *Put1* and *Put2* sequences were directly ligated into the pYES2 vector. Mitochondrial transit peptides in Prodh and P5Cdh were identified with the MitoPro II software version 1.0, and the DNA sequences for these signal peptides were replaced with the *Sdh1*-mTP sequence and fused to the 5′-end of ProdhΔ21 or P5CdhΔ45 by fusion PCR [35]. The final PCR products were cloned into pUC19 for sequence verification before cloning into the pYES2 vector to yield the appropriate complementation constructs, which were transformed into yeast by the LiAc/PEG method [36]. The transformants were selected on a synthetic medium with 20 g/L glucose supplemented with a mixture of amino acids without uracil. For evaluation of growth on organic nitrogen sources, the yeast strains were cultured in 1 mL of YPD medium over night with shaking; the overnight cultures were centrifuged and the resulting pellets were washed once with 1 mL of sterile water and centrifuged again. The pellets were resuspended in 10 μL of sterile water and used to streak on synthetic medium plates with 20 g/L galactose and the specified nitrogen sources.

### Deletion of *Prodh*, *P5Cdh*, and other genes involved in the proline/glutamate pathway

In addition to *Prodh* (JGI protein ID 277618) and *P5Cdh* (JGI protein ID 344979), there are seven other genes involved in the proline/glutamate pathway in *C. parasitica: Pro1* (JGI protein ID 335454), *Pro2* (JGI protein ID 343220), *Pro3* (JGI protein ID 74323), *Car1* (JGI protein ID 99673), *Car2* (JGI protein ID 86840), *Put3* (JGI protein ID 331838), and *Put4* (JGI protein ID 330367). The primer pairs used in the construction of gene disruption mutants for these genes are listed in Table S1. A gene replacement strategy was employed to generate the null mutants. Replacement cassettes were constructed using a double-joint PCR [35] in which the hygromycin B resistance gene *hph* was fused to the 5′- and 3′- flanking fragments of the target genes in a molar ratio of 3:1:1. The PCR reaction cycle consisted of 94°C for 2 min, followed by 15 cycles of 94°C for 30 s, 58°C for 2 min and 72°C for 4 min, with a final extension of 5 min at 72°C. The pUCHyg plasmid was used for the *hph* template DNA and the flanking regions were generated from the genomic DNA of *C. parasitica* by PCR. Next, a nested PCR was performed using the fused DNA as a template; the primers used for the nested PCR are listed in Table S1. Gel electrophoresis-purified RCR products were directly used to transform protoplasts of the *C. parasitica* strain Δ*ku80* [31]. A typical transformation reaction contained 8 × 10^7^ protoplasts in a volume of 100 µL.

The functional complementation of each gene-knockout mutant was performed using a wild-type gene fragment containing a 1.5-kb promoter region, the complete coding region, and a 0.9-kb terminator region, according to an established protocol [37]. Briefly, a full-length target gene was amplified with gene-specific primer sets and cloned into the pUCG418 vector to generate the complementation construct. The construct was then used to transform a mutant null for the gene of interest. The protoplast preparation and transformation were performed as previously described [38], and selection was performed with 30 μg/mL hygromycin or 25 μg/mL neomycin in the regeneration medium. To ensure a true positive, three rounds of screening on selection plates were performed, and the selected transformants were subjected to single-spore purification.

Genomic DNA was extracted from vegetative hyphae as described [39], and a Southern blot analysis was performed with the DIG High Prime DNA Labeling and Detection Starter Kit II from Roche Applied Science (Mannheim, Germany).

### Determination of the intracellular concentration of proline and P5C

The intracellular levels of proline and P5C in mycelia were assayed as previously described for yeast [2]. Briefly, vigorously growing *C. parasitica* was cultured on an agar plate at 25°C for 7 days. The mycelium was collected and dried, and 0.1 g was homogenized in 5 mL of 3% aqueous sulfosalicylic acid and transferred to a boiling water bath for 10 min to extract the intracellular amino acids. The homogenate was filtered through Whatman filter paper. For the determination of the proline content, 2 mL of filtrate was reacted with 2 mL of glacial acetic acid and 2.5 mL of acid-ninhydrin (2.5 g of ninhydrin dissolved in 6 mL of glacial acetic acid and 4 mL of 6 M phosphoric acid) in a Corning tube for 1 h at 100°C. The reaction was stopped by incubation on ice, and the mixture was extracted with 5 mL of toluene. The toluene phase was separated, and the concentration of proline was measured at OD_520_. For the determination of the P5C content, 1 mL of filtrate was added to 0.1 mL of trichloroacetic acid. A volumn of 0.5 mL of 2-AB at 6 mg/ml in 20% ethanol was then added to the mixture, and the reaction was allowed to proceed for 1 h. After centrifugation at 10,000×g for 10 min, the absorbance of the clear supernatant was measured at 443 nm.

### Sporulation

The strains tested were cultured on PDA plates at 25°C for 14 days under laboratory bench-top conditions with a day/night cycle of 12 h/12 h. The conidial spores were collected as a suspension solution in 0.2% Tween 20 and counted under a light microscope with the aid of a hemocytometer [33].

### Virulence assay

Dormant stems of Chinese chestnut (*Castanea mollissima*) were used in virulence assays of the fungal strains with six replicates for each fungal strain. The inoculated stems were kept at room temperature in a plastic bag to maintain moisture, and cankers were measured at 4 weeks after inoculation [37].

## Results

### Identification of genes encoding proline dehydrogenase and P5C dehydrogenase in *C. parasitica*

**Figure 1:**
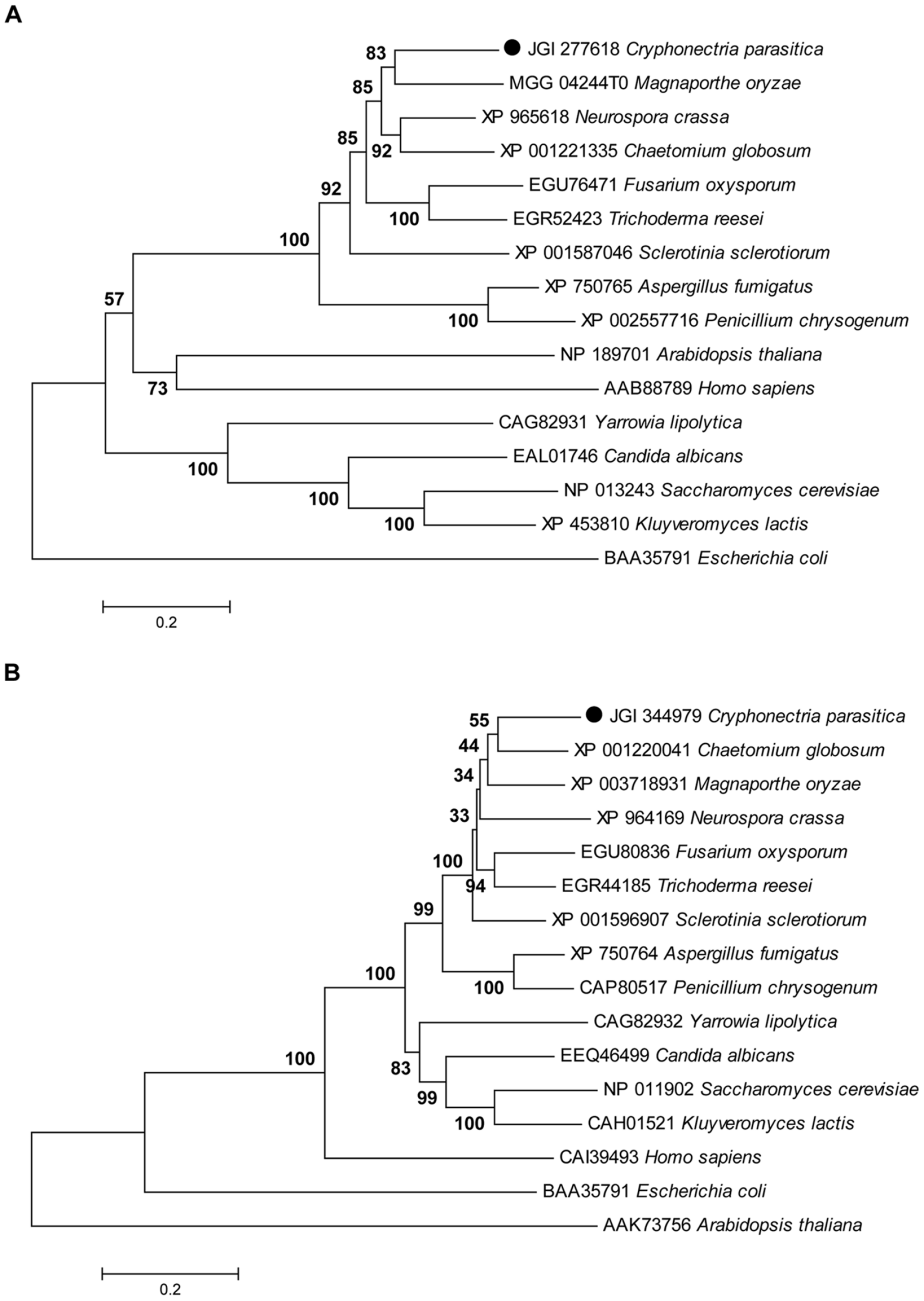
Phylogenetic trees of Prodh and P5Cdh across kingdoms. The phylogenetic trees were generated from amino acid sequences using the Neighbor-joining, minimum-evolution, and maximum-parsimony methods in the MEGA4.0 software. The sequence alignment data were bootstrapped with 1,000 resamplings. The scale bar indicates 0.02 nucleotide substitutions per position. The amino acid sequences of the Prodh and P5Cdh orthologs were downloaded from the NCBI protein database, and accession number for each protein is placed before the name of the protein. A, The tree for Prodhs; B, the tree for P5Cdhs.

Using the complete amino acid sequence of the Put1 proline dehydrogenase (EC 1.5.99.8) enzyme in *S. cerevisiae*, the ortholog in the *C. parasitica* EP155 genome (http://genome.jgi-psf.org/Crypa2/Crypa2.home.html) was identified and designated Prodh. *Prodh* (JGI protein ID 277618) was predicted to encode a 438-aa protein with a predicted molecular mass of 47.6 kDa and a pI of 8.59. A BLASTp search of Prodh against the non-redundant protein sequences (nr) database revealed that it has a conserved ProDH domain (60–434 aa). Prodh in *C. parasitica* shares 65% identity with the putative Prodh of *Magnaporthe oryzae* and 28% identity with Put1 of *S. cerevisiae* (Figure S1A). The phylogenetic tree constructed with Prodh from microbial, plant, and animal sequences showed that the Prodh sequences from ascomycete fungi cluster as a clade, Prodh from plants and humans as a second clade, and Prodh from yeasts a third clade, with *E. coli* Prodh being the most distal clade (Figure 1A). In terms of aa sequence identity, the Prodh proteins are highly diverse among the clades (less than 30% in identity); even in the same clade for ascomycetes, the highest identity is only 65%.

The Put2 ortholog, named P5Cdh in the *C. parasitica* genome was identified using the Put2 aa sequence to blast the genome of *C. parasitica. P5Cdh* (JGI protein ID 344979) was deduced to encode a 594-aa protein with a predicted molecular mass of 64.2 kDa and a pI of 8.65. A BLASTp search of P5Cdh against the nr database revealed that it contains a conserved ALDH_F4-17_P5CDH domain (57–580 aa). P5Cdh shares 66% identity with the putative P5Cdh of *M. oryzae* and 51% identity with Put2 of *S. cerevisiae* (Figure S1 B). The phylogenetic tree constructed with the P5Cdh proteins from microbial, plant, and animal sequences showed a clear lineage, with P5Cdhs from ascomycete fungi clustering as a clade, those from yeasts as a second clade, those from humans as a third clade, those from bacterium as a fourth clade, and those from plants being the most distal clade. Within the ascomycetes, P5Cdhs from *Penicillium* and *Aspergillus* form a sub-clade (Figure 1B). In terms of aa sequence identity, P5Cdhs are less diverse within clades than Prodhs.

### Enzymatic activity of recombinant Prodh and P5Cdh

**Figure 2:**
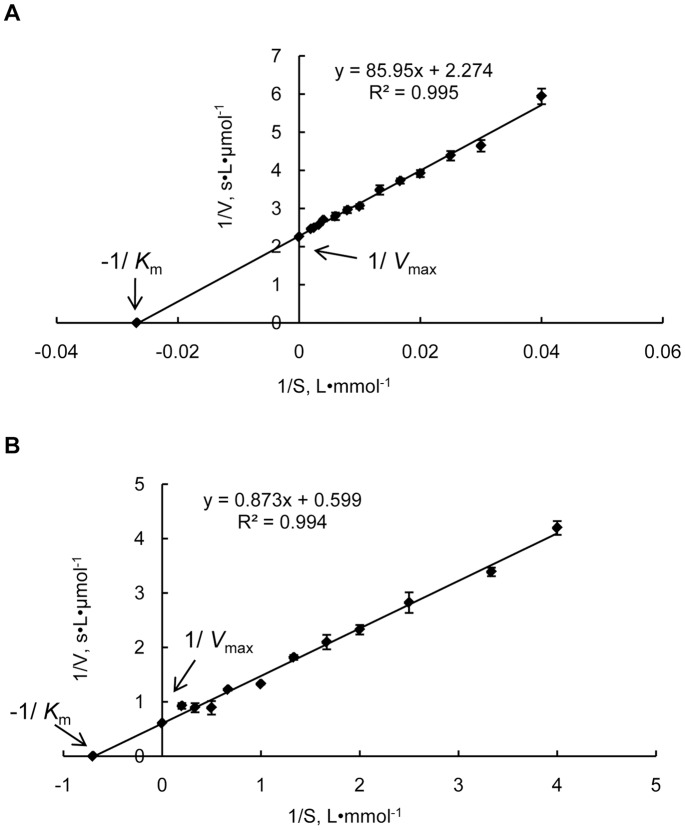
Lineweaver–Burk double reciprocal plots for the purified recombinant proteins GST-ProdhΔ21 and GST-P5CdhΔ45. Steady-state kinetic parameters *V*_max_ and *K*_m_ for the purified recombinant proteins GST-ProdhΔ21 and GST-P5CdhΔ45 were determined by nonlinear regression to fit the data of the Michaelis–Menten equation. The data represent the mean of three independent experiments. A, Prodh activity was assayed based on the dehydrogenation of proline. The reaction velocity was determined at 30°C using 0–500 mM proline as the substrate. The P5C-2-AB concentration was measured at 443 nm; B, P5Cdh activity was assayed based on the dehydrogenation of P5C. The reaction velocity was determined at 30°C using 0–5 mM NAD as the substrate and the electron acceptor. The NADH concentration was measured at 330nm.

cDNAs from JGI protein ID 277618 (hereafter named Prodh) and from JGI protein ID 344979 (thereafter named as P5Cdh) without their mitochondrial targeting sequences were expressed in *E. coli* to generate recombinant proteins GST-ProdhΔ21 and GST-P5CdhΔ45, both with an N-terminal GST tag to facilitate purification. The purified GST-ProdhΔ21 and GST-P5CdhΔ45 proteins were soluble and appeared as clear single bands by SDS-PAGE, with an apparent molecular mass of approximately 72 kDa and 92 kDa, respectively, which matches well with their calculated molecular masses plus the 26-kDa GST-tag. In assays using L-proline as a substrate, the GST-ProdhΔ21 protein displayed typical Michaelis-Menten kinetics, with a maximum enzymatic speed of 0.044±0.010 μmol·s^–1^, a *K*_m_ of 38.15±3.523 mM, and a *k*_cat_ of 0.022±0.005 s^–1^ at 30°C (Figure 2A).

In assays using the P5C as a substrate and NAD as the terminal electron acceptor, GST-P5CdhΔ45 displayed typical Michaelis-Menten kinetics, with a maximum enzymatic speed of 1.69±0.111 μmol·s^–1^, a *K*_m_ of 1.48±0.145 mM, and a *k*_cat_ of 0.076±0.005 s^–1^ at 30°C (Figure 2B).

### *Prodh* and *P5Cdh* can biologically complement yeast *put1* and *put2* mutants

**Figure 3:**
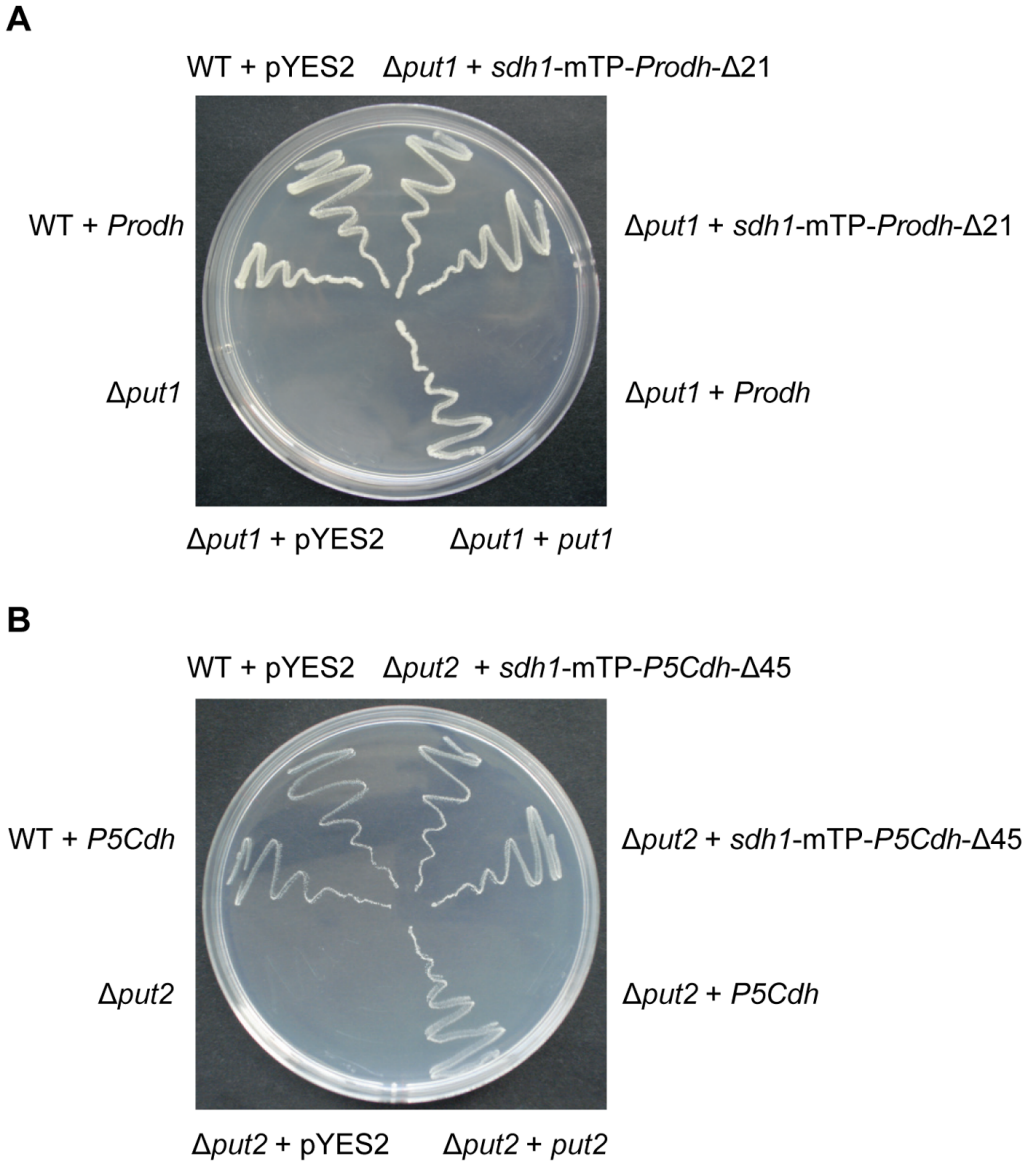
Heterologous complementation of yeast Δ*put1* and Δ*put2* mutants by *Prodh* and *P5Cdh* from *C. parasitica.* The yeast wild-type (WT) and mutant strains transformed with or without complementation constructs were streaked onto minimal medium (MM) with 2% galactose as the carbon source and 10 mM urea or proline as the sole nitrogen source. The WT strain grew well on all media whereas the mutants survived only on 10 mM urea. The mutants were able to grow on MM supplemented with 10 mM proline when the native *Put1* or *Put2* of the yeast or *C. parasitica Prodh* or *P5Cdh* fused to the yeast *Sdh1* mTP was re-introduced into the corresponding mutant. A, Complementation of the yeast Δ*put1* mutant with *Prodh*; B, complementation of the yeast Δ*put2* mutant with *P5Cdh*. The photographs were taken on the 7th day.

The *put1* and *put2* (equivalent to *Prodh* and *P5Cdh*, respectively) null mutants of *S. cerevisiae* cannot grow on a medium with proline as the sole nitrogen source [2]. To test whether the full-length Prodh gene could complement the Put1 mutant, we expressed Prodh in the yeast *put1* yeast mutant 24099. Transformation of the Δ*put1* mutant with pYES2-Prodh, which harbored the full-length cDNA of Prodh, did not enable the cells to metabolize proline. However, after replacing the Prodh mTP (aa 1–21) with that of the flavoprotein subunit of yeast succinate dehydrogenase (Sdh1, aa 1–52), the Prodh fusion was able to restore the proline metabolism to the Δ*put1* strain (Figure 3A). Similarly, expression of P5Cdh with an mTP from Sdh1 enabled the Δ*put2* strain to grow on the medium with proline as the sole nitrogen source (Figure 3B).

### Phenotypic characterization of *Prodh* and *P5Cdh* deletion mutants

**Figure 4:**
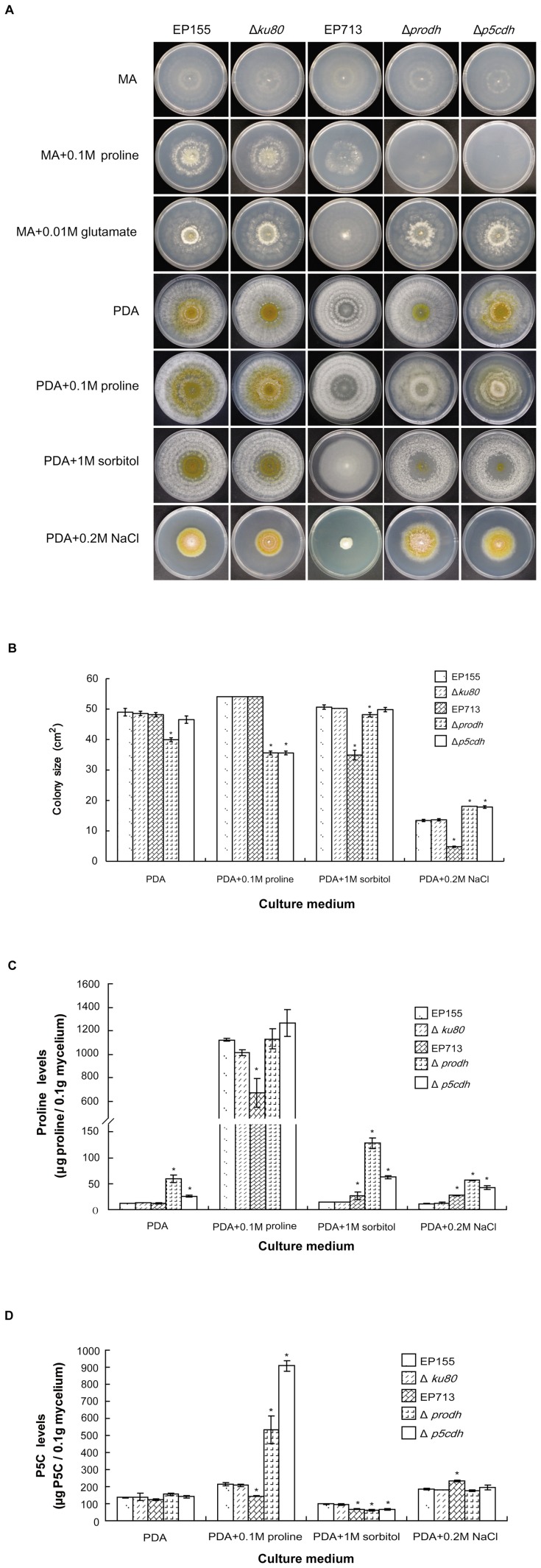
Growth in stressed conditions and intracellular proline and P5C of Δ*prodh* and Δ*p5cdh* mutants. A, Growth on minimal agar (MA) and MA supplemented with proline and glutamate at 25°C for 14 days; growth on PDA and PDA supplemented with proline, sorbitol or NaCl at 25°C for 7 days. B, Colony size of the strains grown on PDA and PDA supplemented with proline, sorbitol or NaCl at 25°C for 7 days. C, Intracellular proline content of the strains cultured on PDA and PDA supplemented with proline, sorbitol or NaCl at 25°C for 7 days. Mycelia were collected from the plates, and the intracellular proline level was assayed according to an established protocol [2]. D, Intracellular P5C content of the strains cultured on PDA and PDA supplemented with proline, sorbitol or NaCl at 25°C for 7 days. Mycelia were collected from the plates, and the intracellular P5C level was measured according to an established protocol [2]. The error bars represent standard deviations. The statistical significance was determined using Student's t-test (*p<0.05). The assays were repeated 3 times.

To explore the effects of *Prodh* and *P5Cdh* deletions on the phenotypic and physiological traits of *C. parasitica, prodh*- and *p5cdh*-null mutants were constructed using gene replacement and confirmed by Southern analyses (Figure S2). A total of 19 Δ*prodh* and 9 Δ*p5cdh* transformants were obtained. Complemented Δ*prodh* and Δ*p5cdh* strains were generated using the wild-type alleles of the *Prodh* and *P5Cdh* genes. The Δ*prodh* mutants were all alike and displayed less orange pigmentation than the wild-type EP155 strain. The Δ*p5cdh* mutants were similar to EP155 but with a slightly intensified orange pigmentation. The complemented *Prodh* mutant (Δ*prodh*-com) and complemented *P5Cdh* mutant (Δ*p5cdh*-com) were indistinguishable from the wild-type strain on PDA (Figure 4A).

### Proline is toxic to Δ*prodh* and Δ*p5cdh* mutants

The deletion of *Prodh* or *P5Cdh* rusults in the loss of the ability to utilize proline as the sole nitrogen source [5]. To test whether *Prodh* or *P5Cdh* perform the same function in the *C. parasitica*, the minimal agar (MA) with glucose as the sole carbon source and without any nitrogen source was used as the base medium with different nitrogen salt supplementation. On MA, all strains grew as a thin layer, with little mycelium mass after 14 days at 25°C (Figure 4A). The growth of the wild-type EP155 strain, the knockout strain Δ*ku80*, and the hypovirus-infected EP713 strain was improved on MA plates when the plates were supplemented with 0.1 M proline; in contrast, the Δ*prodh* and Δ*p5cdh* mutants were not able to grow on MA supplemented with 0.1 M proline. However, when MA was supplemented with glutamate, the Δ*prodh* and Δ*p5cdh* mutants grew as well as the wild-type cells, demonstrating that the poor growth of the Δ*prodh* and Δ*p5cdh* mutants was due to their inability to utilize proline and convert it to glutamate (Figure 4A). All tested strains grew well on PDA, but their response to the addition of extra proline varied: EP155, Δ*ku80*, and EP713 grew better and faster when supplemented with 0.1 M proline than on PDA, but the Δ*prodh* and Δ*p5cdh* mutants grew worse than on PDA (Figure 4B), demonstrating that a higher level of proline was toxic to the Δ*prodh* and Δ*p5cdh* mutants.

### Osmotic response and salt sensitivity of *prodh* and *p5cdh* mutants

When cultured on solid medium with 1 M sorbitol to create osmotic stress, EP155, Δ*ku80*, Δ*prodh*, and Δ*p5cdh* grew at similar rates, with orange pigmentation and conidial spores at the center of the colonies, whereas the Δ*prodh* and Δ*p5cdh* colonies were lush and cotton-like. EP713 showed clear signs of stress under this condition, with a thin and flat colony and a reduced growth rate. Under 0.2 M NaCl stress, all the strains were severely inhibited in growth, with EP713 being the most sensitive, whereas the Δ*prodh* and Δ*p5cdh* mutants out-grew the parental Δ*ku80* and EP155 strains (Figure 4A & B).

### Relationship between *Prodh* and *P5Cdh* and intracellular accumulation of proline and P5C

As shown in Figure 4C, the proline content in the Δ*prodh* mutant was 3 times higher (59 g/0.1 g mycelium) and 1.5 times higher in Δ*p5cdh* mutant (26 g/0.1 g mycelium) than in EP155, Δ*ku80*, and EP713 (12 g/0.1 g mycelium) when cultured on PDA. Using proline production on PDA as a reference, the supplementation of 0.1 M proline increased the intracellular proline to a very high level (660–1200 g/0.1 g mycelium) for all strains, suggesting that proline could be efficiently taken up by the cells. Although 1 M sorbitol did not affect the proline levels in EP155 or Δ*ku80*, the proline levels in EP713, Δ*prodh*, and Δ*p5cdh* significantly elevated (2.0-fold). Supplementation of 0.2 M NaCl did not significantly alter the proline levels relative to the levels produced on PDA for any of the strains, with the exception of EP713 in which the intracellular proline level increased by approximately 2-fold.

The intracellular P5C levels were similar (130 g/0.1 g mycelium) for all the strains on PDA. Using PDA as a reference, P5C in the Δ*prodh* strain increased from 156 to 533 g/0.1 g mycelium and accumulated to an even higher level in the Δ*p5cdh* strain, from 142 to 907 g/0.1 g mycelium, when PDA was supplemented with 0.1 M proline (Figure 4D).

The addition of 1 M sorbitol slightly decreased P5C accumulation in EP155 and Δ*ku80* but significantly decreased it (from 130 to 60 g/0.1 g mycelium) in EP713, Δ*prodh*, and Δ*p5cdh*. The addition of 0.2 M NaCl elevated the intracellular P5C levels for all the strains by approximately 60–70% (Figure 4D).

### Prodh and P5Cdh are responsible for intracellular Prodh and P5Cdh activities

In an assay for proline dehydrogenase activity using cell extracts, the Δ*prodh* mutant exhibited a very low level of proline dehydrogenase activity, approximately 1/10 of the activity observed in the parent strain Δ*ku80* under various culture conditions, confirming that Prodh is the major enzyme responsible for proline dehydrogenation in the cells (Table 1). When grown on PDA, the deletion of *P5Cdh* resulted in significantly lower Prodh activity (40% of Δ*ku80*), suggesting that P5Cdh has a feedback influence on Prodh (Table 1). It was also noted that the Prodh activity in EP713 was significantly lower than in EP155 (16% of EP155). The Prodh activity was induced by 3-8-fold for all strains (except the Δ*prodh* mutant) when proline was added to the medium, with the Prodh activity in Δ*p5cdh* increasing the most (8-fold). Although the enzymatic activity in EP713 increased with the supplementation of external proline, the activity only accounted for 25% of that in EP155. The addition of 1.0 M sorbitol or 0.2 M NaCl to the medium significantly suppressed the Prodh activity in all strains.

**Table 1:** Intracellular Prodh activity of *C. parasitica* strains in various culture media^a^.

	PDA	PDA+0.1M proline	PDA+1M sorbitol	PDA+0.2M NaCl
EP155	0.425±0.059	1.218±0.072	0.257±0.018	0.238±0.068
Δ*ku80*	0.249±0.053	0.824±0.134	0.239±0.012	0.281±0.063
EP713	0.069±0.006	0.313±0.044	0.238±0.005	0.135±0.023
Δ*prodh*	0.024±0	0.028±0.006	0.027±0.016	0.018±0.004
Δ*p5cdh*	0.099±0.024	0.862±0.069	0.078±0.008	0.114±0.009

a Prodh activity was expressed as 1 μM of P5C formed per min per 100 mg of mycelium. The data are from three replicates.

Similar assays were performed for the P5C dehydrogenase activity, which was very low in the Δ*p5cdh* mutant compared to the parental strain Δ*ku80* under all the tested conditions, confirming that P5Cdh is the major enzyme for P5C dehydrogenation in *C. parasitica*. As shown in Table 2, deletion of *Prodh* did not significantly affect the accumulation of P5C in the cells, nor did a hypovirus infection (EP713). The addition of exogenous 0.1 M proline to PDA significantly induced P5C dehydrogenase activity in all the strains except the Δ*p5cdh* mutant. The supplementation of 1 M sorbitol or 0.2 M NaCl did not substantially influence the P5C levels in any of the strains (Table 2).

**Table 2:** Intracellular P5Cdh activity of *C. parasitica* strains in various culture media^a^.

	PDA	PDA+0.1M proline	PDA+1M sorbitol	PDA+0.2M NaCl
EP155	1.993±0.074	3.304±0.091	1.826±0.154	1.992±0.249
Δ*ku80*	1.831±0.14	4.396±0.269	1.379±0.051	1.783±0.326
EP713	1.594±0.016	3.421±0.131	1.817±0.135	1.889±0.153
Δ*prodh*	1.16±0.067	3.847±0.081	1.402±0.124	1.843±0.205
Δ*p5cdh*	0.138±0.019	0.194±0.027	0.135±0.014	0.178±0.004

a P5Cdh activity was expressed as 1 μM of NADH formed per min per 100 mg of mycelium. The data are from three replicates.

### Requirement of proline/glutamate pathway components for virulence and sporulation

**Figure 5:**
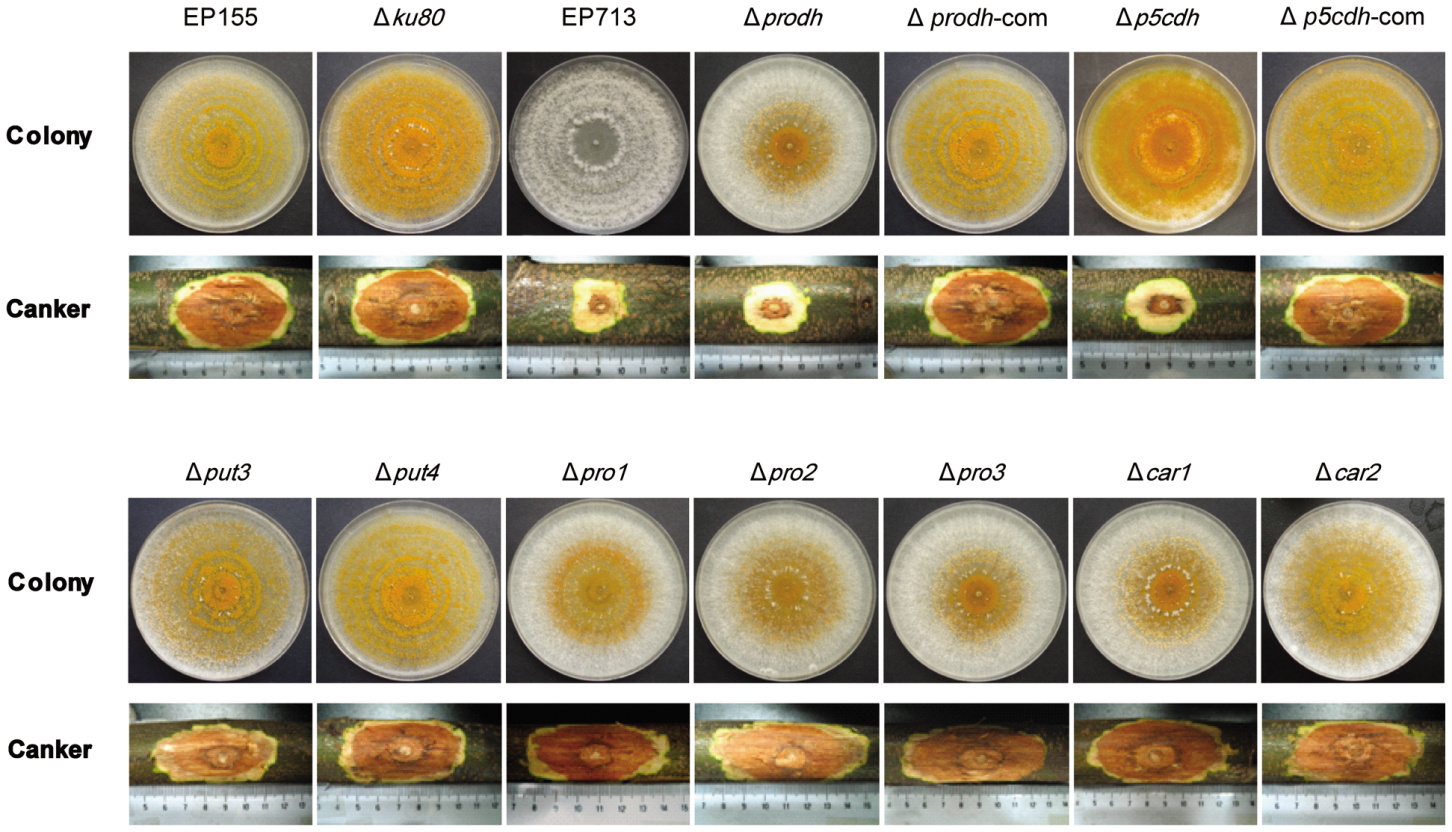
Colony morphology and virulence assay of mutant strains. The strains were cultured on PDA at 25°C and photographs were taken on the 14th day. Virulence assays were performed on dormant stems of Chinese chestnut (*Castanea mollissima*). The inoculated stems were kept at room temperature in a plastic bag to maintain moisture, and the cankers were measured at 4 weeks post-inoculation and photographed. The assays were performed with six replicates per fungal strain.

To evaluate the contribution of the components of the proline/glutamate pathway to the virulence and conidial spore production of *C. parasitica*, genes encoding the components of the proline/glutamate pathway were individually knocked out, and the mutants were assayed for virulence by inoculation on the dormant chestnut stems. As shown in Figure 5, all the mutants of the proline/glutamate pathway were able to grow on PDA, demonstrating that none of these genes are essential for saprophytic growth. Although the Δ*car1*, Δ*car2*, Δ*pro1*, Δ*pro2*, Δ*pro3*, Δ*put3*, and Δ*put4* mutants caused normal cankers on the chestnut stems, the deletion of *Prodh* or *P5Cdh* significantly impaired the ability of the mutants to grow on chestnut stems to the same level as the hypovirus-infected strain EP713 (Figure 5, Table 3). When the level of sporulation was determined, it was found that the deletion of *Pro1, Pro2*, or *Prodh* suppressed sporulation by 1 to 2 orders of magnitude compared to the parental strain, suggesting that the regulation of virulence and sporulation is not coupled (Table 3).

**Table 3:** Characterization of mutant strain sporulation and virulence.

Strain	Conidial spores/mL^a^	Canker area (cm^2^)^b^
EP155	2.08×10^7^±3.38×10^6^	9.85±0.47
Δ*ku80*	1.79×10^7^±1.63×10^6^	8.97±1.00
EP713	0	1.27±0.52*
Δ*prodh*	1.76×10^6^±1.65×10^5^*	1.78±0.85*
Δ*p5cdh*	2.51×10^7^±1.03×10^6^	1.58±0.85*
Δ*put3*	2.27×10^7^±2.23×10^6^	9.95±0.74
Δ*put4*	2.36×10^7^±3.13×10^6^	10.57±0.90
Δ*pro1*	1.16×10^6^±4.17×10^5^*	9.25±0.84
Δ*pro2*	1.01×10^5^±5.65×10^4^*	8.99±0.51
Δ*pro3*	1.33×10^7^±1.44×10^6^	9.55±0.89
Δ*car1*	1.67×10^7^±3.13×10^6^	9.07±1.02
Δ*car2*	1.76×10^7^±3.13×10^6^	8.55±0.95

a Conidiation was measured after culturing on PDA plates at 25°C for 14 days. The mean and standard deviation were calculated from three replicates. *indicates a statistically significant difference (p<0.05).

b The virulence assays were performed on dormant chestnut stems. The inoculated stems were kept at 25°C for 30 days. The mean and standard deviation were calculated from three replicates. *indicates a statistically significant differences (p<0.05).

### Hypovirus suppresses the expression of *Prodh* and *P5Cdh*

**Figure 6:**
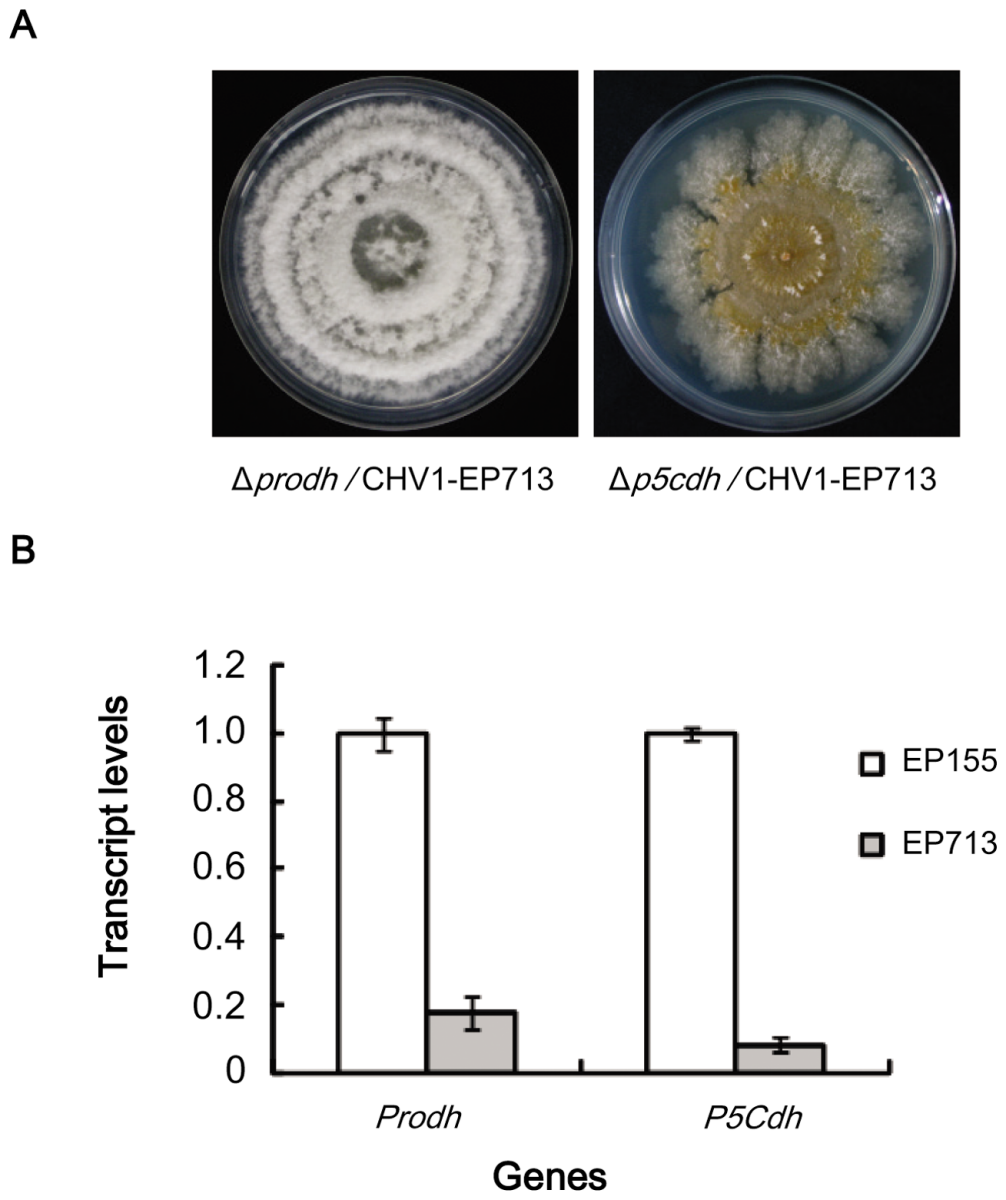
Impact of hypovirus infection on the morphology of Δ*prodh* and Δ*p5cdh* and on the accumulation of *Prodh* and *P5Cdh* transcripts in the wild-type strain. A, Morphology of hypovirus-infected Δ*prodh* and Δ*p5cdh* mutants. The strains were cultured on PDA for 7 days; hypovirus was introduced into Δ*prodh* and Δ*p5cdh* from the hypovirus-infected strain EP713 by anastomosis. Paired inoculation of the Δ*prodh* or Δ*p5cdh* mutant with EP713 resulted in anastomosis and the conversion of the strains to the hypovirus infection phenotype. B, Transcript accumulation levels in hypovirus-free EP155 and hypovirus-infected EP713. Fungal strains were cultured on PDA at 25°C for 7 days, and mycelia were collected for mRNA isolation. The transcript levels were determined by RT-PCR using the *Prodh*-specific primer pair prodh-Qf/prodh-Qr and *P5Cdh*-specific primer pair p5cdh-Qf/p5cdh-Qr. The transcript level in EP713 is expressed as a percentage of the transcript level in EP155. The values were calculated from three independent experiments. The error bars represent standard deviations.

The paired inoculation of the hypovirus-infected strain EP713 with either the Δ*prodh* or Δ*p5cdh* mutant resulted in anastomosis and the conversion of the mutant strains to the hypovirus infection phenotype, as characterized by the loss of orange pigmentation and suppressed sporulation. The virus-containing colony of Δ*prodh*/CHV1-EP713 was visually similar to EP713 but with fluffier and thicker aerial hyphae. In contrast, the virus-containing colony of Δ*p5cdh*/CHV1-EP713 grew slowly, with irregular edges and intensified orange pigmentation (Figure 6A). Although the mutants infected with virus appeared to have different phenotypes, the viral dsRNA accumulation in each mutant was comparable with that observed in EP713 (data not shown), suggesting that the deletion of either *Prodh* or *P5Cdh* did not have an impact on hypovirus replication or maintenance. However, an examinaion of transcript levels revealed that the accumulation of *Prodh* and *P5Cdh* transcripts was down-regulated by 5- and 10-fold, respectively, in the infected strain compared to the wild-type (Figure 6B).

## Discussion

### Evolutionary relationship between Prodh and P5Cdh across kingdoms

Prodh is characterized by a ProDH domain (Figure S1A) [40], and enzymatic assays verified the Prodh activity of JGI 277618 in *C. parasitica* (Figure 2A). A similar approach lead to the identification of P5Cdh (JGI 344979) in *C. parasitica* having P5Cdh activity (Figure S1B; Figure 2B). Though not many Prodh enzymes or genes have been characterized in fungi, the deduced Prodh amino acid sequences available in GenBank and other databases allow for the comparison of Prodh among microbial, plant, and animal sequences. As illustrated in Figure 1, Prodh from filamentous ascomycetes cluster into a clade, whereas those from yeasts, plants, and animals form their own clades, with Prodh from the bacterium *E. coli* forming a distal clade. Within filamentous ascomycetes, Prodh from *C. parasitica* has 65% identity with Prodh from *M. oryzae*; however, the Prodh identity is only 28% between *C. parasitica* and *S. cerevisiae* and 23.6% between *S. cerevisiae* and *Arabidopsis thaliana* [41]. The high divergence among Prodhs suggests that the evolution of this enzyme began a long time ago. Because Prodh functions in mitochondria, it is likely that this enzyme originated from an ancient bacterium [42].

In contrast to Prodh, P5Cdhs from the species of different kingdoms are relatively more conserved, with identities of 50% or more at the amino acid level (Figure S1B). Our phylogenetic tree shows that there is no clear-cut lineage for this enzyme in species of filamentous ascomycetes, whereas we found clearly defined clades among filamentous ascomycetes, yeasts, humans, bacteria, and plants, indicating that this enzyme may have diverged in a somewhat different way from Prodh (Figure 1B). Prodh and P5Cdh are present in both saprophyte (*Neurospora crassa*) and pathogenic fungi (*C. parasitica, M. oryzae* and *Fusarium oxysporum*), suggesting that these enzymes perform at least some shared basic cellular functions.

### Conserved biological functions of Prodh and P5Cdh

In yeast, intracellular proline confers stress tolerance to freezing, desiccation, oxidation stress, and ethanol stress [5], [43], [44]. However, few studies using other fungi have been reported with regard to Prodh and P5Cdh. Unlike the limited studies in fungi, proline dehydrogenase has been extensively studied in plants. In *A. thaliana*, two *ProDH* genes have been identified and functionally characterized. *ProDH1* is a dehydration-responsive gene and is up-regulated after rehydration, accompanied by a decrease of intracellular proline [41]. *ProDH1* appears to be the dominant isoform under most conditions and in most tissues, whereas *ProDH2* is specifically up-regulated during salt stress [45]. Proline functions to protect plants from drought and salinity stress [46], and ProDH is one of the key enzymes that regulates proline accumulation *in vivo* [47]. However, proline is toxic to cells by playing a negative role in the repression of normal morphogenesis in *Arabidopsis* [48]. The similar negative effect of excessive proline could also arise from P5C, the catalytic product of proline by Prodh; excessive proline and P5C are toxic to cells because they induce ROS accumulation and programmed cell death [8]. There are also two *ProDH* genes in tobacco, *NtPDH1* and *NtPDH2*, which respond to and regulate proline metabolism during drought stress and subsequent recovery [49]. *NtPDH1* is less sensitive to dehydration or rehydration, whereas *NtPDH2* responds rapidly to both conditions and is down-regulated under drought. Proline toxicity has been observed upon mutation of the *Arabidopsis* ortholog of P5Cdh [50]. The external application of proline caused the accumulation of P5C and programmed cell death, and proline and P5C/glutamate semialdehyde have been suspected to serve as a link between stress responses and cell death [51].

In humans, proline dehydrogenase, named POX, is induced by p53 and can regulate cell survival and mediate programmed cell death by causing G_2_ cell cycle arrest to reduce tumor formation and increase the production of α-ketoglutarate to impair HIF-1α signaling [52]. POX is up-regulated by oxidized low-density lipoproteins through peroxisome proliferator-activated receptor gamma and plays a key role in the regulation of protective autophagy in cancer cells [53]. It has been reported that mutation of the *P5Cdh* ortholog *ALDH4A1* in humans causes the genetic disease type II hyperprolinemia, which is characterized by elevating levels of P5C, resulting in mental retardation and convulsions [54].

Despite the diverse biological functions of Prodh and P5Cdh in various species, the fact that Prodh and P5Cdh from a plant were able to function in yeast [41], [45], [50] suggests some conserved basic functions of these enzymes across species in different kingdoms. Although they have low sequence identity, *Prodh* and *P5Cdh* from *C. parasitica* were able to complement the *put1* and *put2* mutants of *S. cerevisiae* (Figure 3), demonstrating these two proteins have the same *in vivo* functions. The fact that both the Δ*prodh* and Δ*p5cdh* mutants were able to grow as well as EP155 and Δ*ku80* on the minimal agar with 0.01 M glutamate as the sole nitrogen source confirms that the inability to convert proline to glutamate is the cause of the alteration in the phenotypes exhibited by the Δ*prodh* and Δ*p5cdh* mutants. Similar to the yeast, the deletion of either *Prodh* or *P5Cdh* increases resistance to salt stress with an increase in proline (Figure 4).

An interesting observation from this and previous reports is that, for proper function, the heterologous Prodh or P5Cdh proteins must be fused with the mTP from yeast to direct the correct localization of the enzymes to the mitochondrial membrane (Figure 3) [41], [45], [50]. Thus, mTP might serve as a species-specific tag for Prodh and P5Cdh from different species.

### Implication of the proline/glutamate pathway and mitochondrial function in the regulation of virulence and sporulation

**Figure 7:**
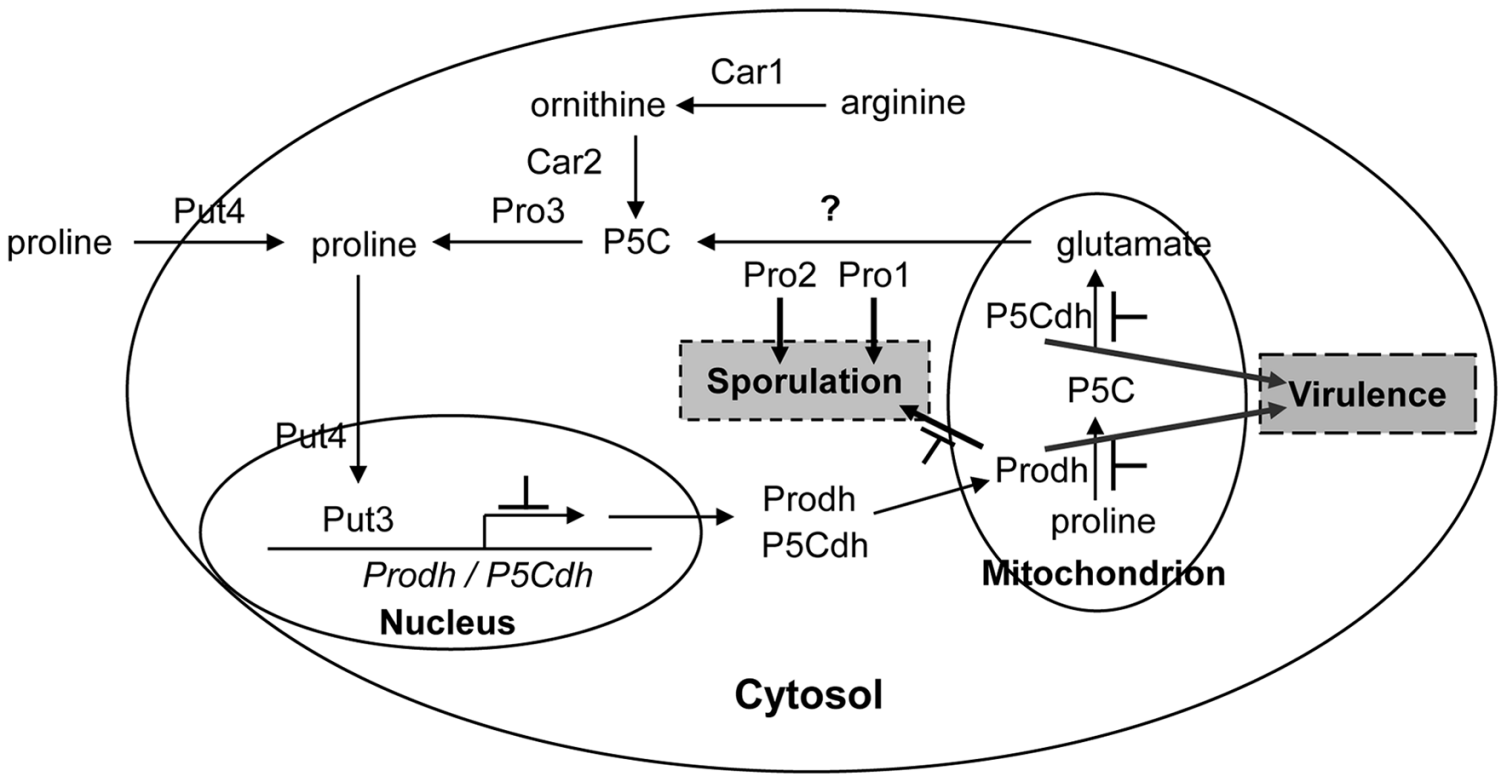
A model of hypovirus regulation of the proline/glutamate pathway. Proline can either be taken up into the cells from the environment by Put4 [59], or generated in the cell by the conversion of P5C by Pro3. Proline in the cytosol induces the expression of Put3 [56], which activates the transcription of *Prodh* and *P5Cdh*. Prodh and P5Cdh translocate into the mitochondria where to catalyze glutamate biogenesis; glutamate can be converted to proline via P5C when it is transported to the cytosol. The conversion of glutamate to P5C can be catalyzed by Pro1 and Pro2 [58], and P5C can then be converted to proline by P5C reductase Pro3 [57]. If the intracellular glutamate levels are insufficient for proline synthesis, proline biogenesis can be initiated from arginine, as catalyzed by Car1 (arginase), to yield ornithine; ornithine is then converted to P5C by Car2 [55]. In this network, P5C and glutamate appear to be vitally important for both virulence and sporulation. By suppressing *Prodh* and *P5Cdh* expression, the hypovirus blocks the biogenesis of P5C and glutamate in mitochondria, resulting in hypovirulence and suppressed sporulation. Although Pro1 and Pro2 do not appear to be regulated by the hypovirus at the transcriptional level, the possibility that they might be regulated at the protein level can not be ruled out, as hypovirus-encoded proteins have been detected in mitochondria [65].

Proline catabolism in the cell involves both cytosol and mitochondria. The conversion of glutamate to proline by P5CS and P5CR and the conversion of arginine to proline by Car1 and Car2 occur in the cytosol, whereas the conversion of proline to glutamate by Prodh and P5Cdh occurs in the mitochondria (Figure 7) [55]–[59]. The deletion of genes encoding P5CS, P5CR, Car1, and Car2 did not affect the virulence (Figure 5), though deletion of *Prodh* and *P5Cdh* resulted in hypovirulence (Figure 5), demonstrating that the *prodh*- and *p5cdh*-associated mitochondrial functions are required in the regulation of virulence. This is the first report demonstrating that proline catabolism and glutamate biogenesis are indispensible for virulence in a pathogenic fungus.

Although both *Prodh* and *P5Cdh* are required for virulence, only *Prodh* is required for sporulation (Table 3), demonstrating that virulence and sporulation are two different processes and that Prodh is a multifunctional protein. A closer inspection of the components of the proline/glutamate pathway also revealed that *Pro1* and *Pro2* are required for proper sporulation (Table 3). As the gene products of *Pro1* and *Pro2* function to convert glutamate to P5C, it is suspected that a proper level of P5C is required for the formation of conidial spores in *C. parasitica*.

A mutation in the mitochondrial DNA (mtDNA) of the *C. parasitica* strain EP155/mit2 was previously shown to cause hypovirulence, with the production of very few asexual spores and an elevated alternative oxidase activity [29]. The fact that proline is toxic to Δ*prodh* and Δ*p5cdh* mutants and that P5C accumulated in the cell after proline supplementation (Figure 4) correlated to the elevated oxidase activity found in the EP155/mit2 strain, as P5C would lead to ROS accumulation, which is generated by oxidase [51], [60]. In this regard, the evidence reported in this work suggests that the mitochondrial dysfunction for hypovirulence and hypovirulence-associated traits (i.e. suppressed sporulation) could be due to the malfunction of a physiological process, i.e., proline/glutamate pathway impairment (Figure 7).

It has been reported that when the rice blast fungus *M. oryzae* was cultured on a medium in the absence of preferential nitrogen sources, a number of nitrogen metabolism genes, such as *NPR2* and *MPG1*, were up-regulated. Furthermore, mutation in one or more of these genes resulted in the failure of the fungus to grow under nitrogen starvation conditions and in the loss of pathogenicity [61]. *Prodh* in *M. oryzae* was reported to be up-regulated 8.2- and 3.9-fold after 12 h and 48 h, respectively, during nitrogen starvation, suggesting its involvement in nitrogen utilization [62]. Thus, the inability of the Δ*prodh* and Δ*p5cdh* mutants to incite virulence cankers on dormant chestnut stems could be partly attributed to their inability to synthesize or acquire sufficient glutamate *in planta*.

### Mechanisms of hypovirus perturbation of mitochondrial function

Although mutations in the *C. parasitica Prodh* and *P5Cdh* genes did not appear to have an impact on the accumulation of hypovirus dsRNA, viral infection did cause profound phenotypic changes to the mutants: Δ*prodh*/CHV1-EP713 had similar colony morphology as EP713, but Δ*p5cdh*/CHV1-EP713 showed a distinct phenotype (Figure 6A). The variation in the response to viral infection suggests a possible virus/host interaction at the levels of these gene products. The fact that a hypovirus infection profoundly down-regulates the transcription of *Prodh* and *P5Cdh* (Figure 6B) implies that there would be a shortage of Prodh and P5Cdh in the mitochondria of the host cell. Indeed, Prodh activity in EP713 was significantly lower than in the virus-free isogenic strain EP155 (Table 1). It is anticipated that insufficient Prodh and P5Cdh would jeopardize the energy supply of the cell and hamper the normal biological process of the host, including virulence and sporulation.

In contrast to its low transcript accumulation, P5Cdh activity in the cell extract assays, was the same in EP713 as in the virus-free strain (Table 2). This discrepancy is possibly due to the method used for assaying P5Cdh activity, i.e. measuring the conversion rate of NAD into NADH. We noted that the NADH dehydrogenase, capable of reducing NAD to NADH, was up-regulated 6-fold in the hypovirus-infected *C. parasitica* starin EP713 (our unpublished data).

Although the suppression of *Prodh* and *P5Cdh* expression may be a mechanism by which a hypovirus perturbs the mitochondrial function, this does not rule out the possibility of a direct effect of viral protein on the mitochondria. ORF A of the hypovirus CHV1-EP713 has been shown to suppress sporulation and orange pigmentation when it was transformed into a virus-free fungus strain [63]. It was later found that p29 of the polyprotein encoded by ORF A is responsible for suppressed sporulation and orange pigment production [64]. Moreover, p29 was very recently detected in mitochondria [65]. Thus, it is speculated that p29 may adversely regulate mitochondrial function by interacting with key components of the mitochondrion, of which Prodh, P5Cdh, Pro1, and Pro2 could be good candidates.

Perturbation of the trimeric G-protein signaling pathway [16]–[20] and MAPK signaling pathway [16], [23]–[26] by the hypovirus largely contributes to the multi-faceted alteration in *C. parasitica* phenotypes. Indeed, these pathways cover a large range of cell processes, from sensing environmental cues to the execution of cellular functions. In contrast, only a few downstream targets that execute an exact biochemical function have been reported [27], [37], [66], [67]. In this regard, the unveiling of viral perturbation of the proline/glutamate pathway in this work adds new knowledge to this colletion. Although the deletion of *Prodh* did not appear to perturb the trimeric G-protein and MAPK signaling pathways at the transcriptional level (data not shown), the transcript levels of *Oah1*, a gene encoding the hydrolase Oah1 that hydrolyzes oxaloacetate to produce oxalic acid, were significantly down-regulated in the Δ*prodh* mutant and in the hypovirus-infected strain EP713 (Figure S3). It has been reported that Oah1 is a virulence factor in both humans and plant pathogenic fungi [68]–[71]. Its transcriptional down-regulation in the Δ*prodh* mutant and in EP713 suggests that *Oah1* expression was positively regulated by *Prodh* and suppressed by hypovirus infection. Because the suppression of *Oah1* expression was much more severe in the hypovirus-infected cells than in the *Prodh* mutant, it is reasonable to conclude that in addition to regulating *Prodh*, hypoviruses may also regulate *Oah1* via a different mechanism.

## Supporting Information

Figure S1 **Alignment of Prodh and P5Cdh from** ***C. parasitica*** **,** ***M. oryzae*** **, and** ***S. cerevisiae.*** The amino acid sequences of Prodh and P5Cdh from *C. parasitica, M. oryzae* and *S. cerevisiae* were identified by searching against NCBI genomic BLAST databases and were downloaded from the NCBI protein database. Alignment of the amino acid sequences was performed using the alignment program in Vector NTI 11.0. A. The alignment of Prodh revealed that Prodh shares 65% and 28% identity with the putative protein of *M. oryzae* (MGG_04244T0) and Put1 of *S. cerevisiae* (NP_013243.1), respectively. The red bold lines indicate the conserved domains of ProDH. Identical amino acids are shaded in yellow, and blocks of similarity in green. B, The alignment of P5Cdh revealed that P5Cdh shares 66% and 51% identity with the putative protein of *M. oryzae* (EHA49347.1) and the Put2 of *S. cerevisiae* (AAB68907.1), respectively. The red bold lines indicate the conserved domains of ALDH_F4-17_P5CDH domains. Identical amino acids are shaded in yellow, and blocks of similarity in green.
(TIF)

Figure S2 **Strategy for construction and confirmation of the knock-out mutants.** A, Strategy for the construction of the Δ*prodh* mutant. The *Prodh* gene structure and positions of primers used to generate the gene replacement cassette are shown at the top. An 885-bp fragment at the 5′ end and a 905-bp fragment at the 3′ end of *Prodh* were amplified by PCR. A hygromycin resistance gene cassette was used to replace the complete coding region and a portion of the 3′ UTR of the *Prodh*. B, Strategy for the construction of the Δ*p5cdh* mutant. The *P5Cdh* gene structure and positions of the primers used to generate the gene replacement cassette are shown at the top. A 974-bp fragment at the 5′ end and a 1038-bp fragment at the 3′ end of *P5Cdh* were amplified by PCR. The hygromycin resistance gene cassette was used to replace the largest exon of *P5Cdh* near the 3′ end. C, Southern blot analysis of the *prodh* null mutant. Δ*prodh* was developed from Δ*ku80*, which was derived from the wild-type strain EP155. Restriction digest with *Bgl*II released a 1.9 kb 3′ flanking region of *Prodh* from the wild-type and a 4.1 kb fragment containing the 3′ flanking region from the Δ*prodh*. Probe 1 hybridized with the 3′ flanking region of *Prodh*, and probe 2 recognized the *trpC* promoter carried in the transformation vector cassette. D, Southern blot analysis of the Δ*p5cdh* null mutant. Restriction digest with *Hind*III released a 3.5 kb 5′ region of *P5Cdh* from the wild-type and a 4.2 kb DNA fragment containing the 5′ region of *P5Cdh* from *P5Cdh* null mutant. Probe 3 hybridized to the 5′ flanking region of *P5Cdh*, and probe 2 recognized the *trpC* promoter carried in the transformation vector cassette.
(TIF)

Figure S3 **Quantification of the transcript level of** ***Oah1.*** The strains were cultured on PDA at 25°C for 7 days, and mycelia were collected for mRNA isolation. The *Oah1* transcript accumulation levels were determined by RT-PCR using the *Oah1*-specific primers oah1-Qf and oah1-Qr. The transcript level in EP155 was set at 1.0, and the corresponding levels in the other strains are expressed as a percentage of the levels in EP155. The values were calculated from three independent experiments. The error bars represent standard deviations.
(TIF)

Table S1 **Primers used in this work.**
(DOC)
