# Supplementary figures and images for: Δ1-Pyrroline-5-Carboxylate/Glutamate Biogenesis Is Required for Fungal Virulence and Sporulation
Source: PLoS One. 2013 Sep 9;8(9):e73483. doi: 10.1371/journal.pone.0073483 (PMC3767830; doi:10.1371/journal.pone.0073483)

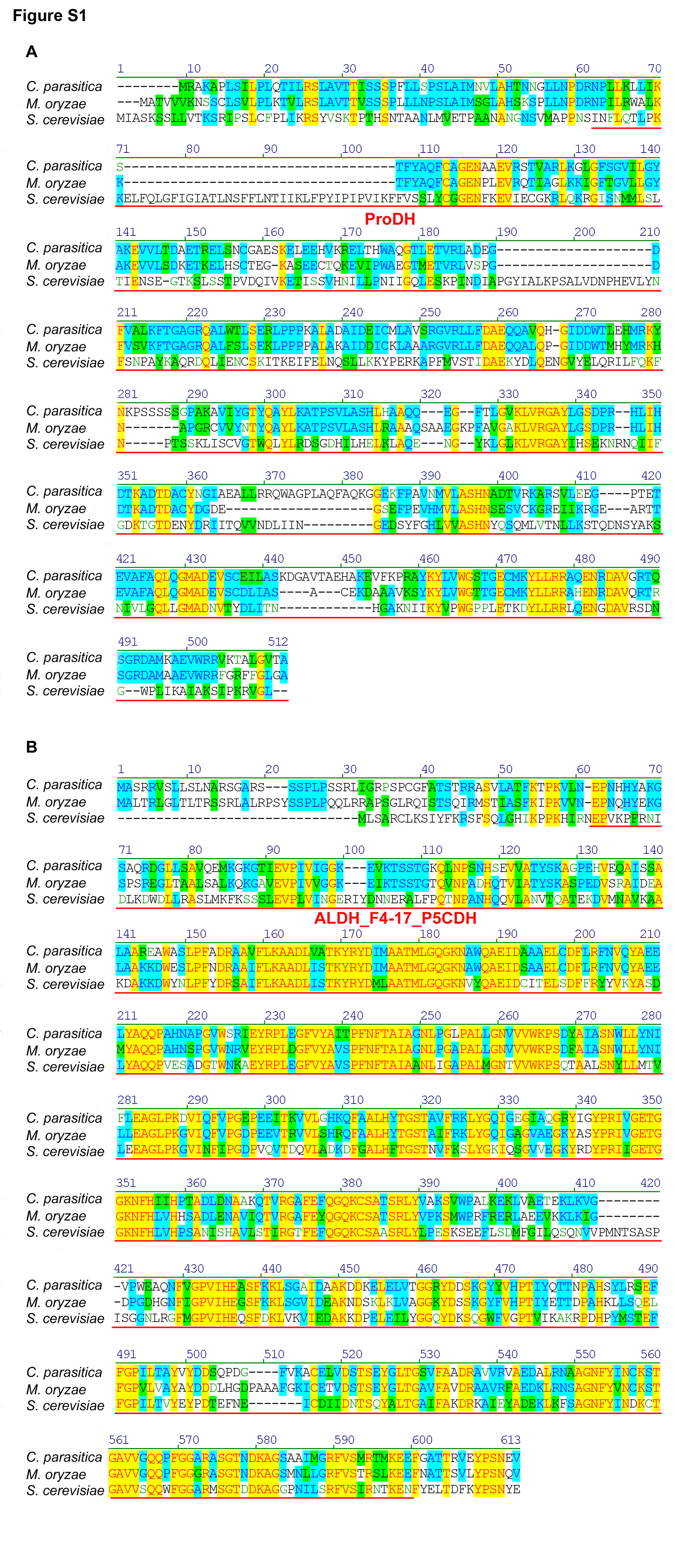

Supplement: Figure S1 — Alignment of Prodh and P5Cdh from C. parasitica , M. oryzae , and S. cerevisiae. The amino acid sequences of Prodh and P5Cdh from C. parasitica, M. oryzae and S. cerevisiae were identified by searching against NCBI genomic BLAST databases and were downloaded from the NCBI protein database. Alignment of the amino acid sequences was performed using the alignment program in Vector NTI 11.0. A. The alignment of Prodh revealed that Prodh shares 65% and 28% identity with the putative protein of M. oryzae (MGG_04244T0) and Put1 of S. cerevisiae (NP_013243.1), respectively. The red bold lines indicate the conserved domains of ProDH. Identical amino acids are shaded in yellow, and blocks of similarity in green. B, The alignment of P5Cdh revealed that P5Cdh shares 66% and 51% identity with the putative protein of M. oryzae (EHA49347.1) and the Put2 of S. cerevisiae (AAB68907.1), respectively. The red bold lines indicate the conserved domains of ALDH_F4-17_P5CDH domains. Identical amino acids are shaded in yellow, and blocks of similarity in green. (TIF) [file pone.0073483.s001.tif]

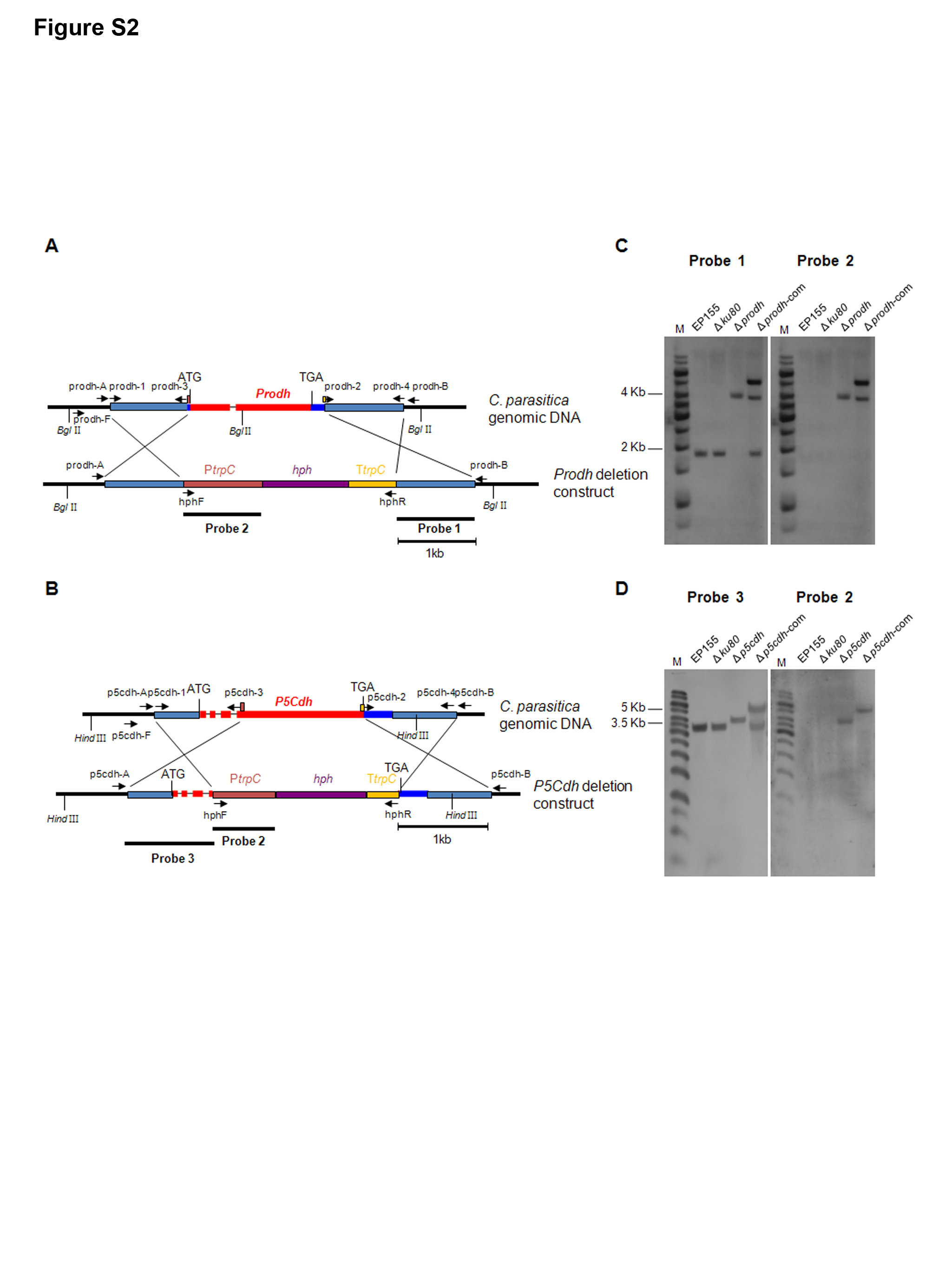

Supplement: Figure S2 — Strategy for construction and confirmation of the knock-out mutants. A, Strategy for the construction of the Δprodh mutant. The Prodh gene structure and positions of primers used to generate the gene replacement cassette are shown at the top. An 885-bp fragment at the 5′ end and a 905-bp fragment at the 3′ end of Prodh were amplified by PCR. A hygromycin resistance gene cassette was used to replace the complete coding region and a portion of the 3′ UTR of the Prodh. B, Strategy for the construction of the Δp5cdh mutant. The P5Cdh gene structure and positions of the primers used to generate the gene replacement cassette are shown at the top. A 974-bp fragment at the 5′ end and a 1038-bp fragment at the 3′ end of P5Cdh were amplified by PCR. The hygromycin resistance gene cassette was used to replace the largest exon of P5Cdh near the 3′ end. C, Southern blot analysis of the prodh null mutant. Δprodh was developed from Δku80, which was derived from the wild-type strain EP155. Restriction digest with BglII released a 1.9 kb 3′ flanking region of Prodh from the wild-type and a 4.1 kb fragment containing the 3′ flanking region from the Δprodh. Probe 1 hybridized with the 3′ flanking region of Prodh, and probe 2 recognized the trpC promoter carried in the transformation vector cassette. D, Southern blot analysis of the Δp5cdh null mutant. Restriction digest with HindIII released a 3.5 kb 5′ region of P5Cdh from the wild-type and a 4.2 kb DNA fragment containing the 5′ region of P5Cdh from P5Cdh null mutant. Probe 3 hybridized to the 5′ flanking region of P5Cdh, and probe 2 recognized the trpC promoter carried in the transformation vector cassette. (TIF) [file pone.0073483.s002.tif]

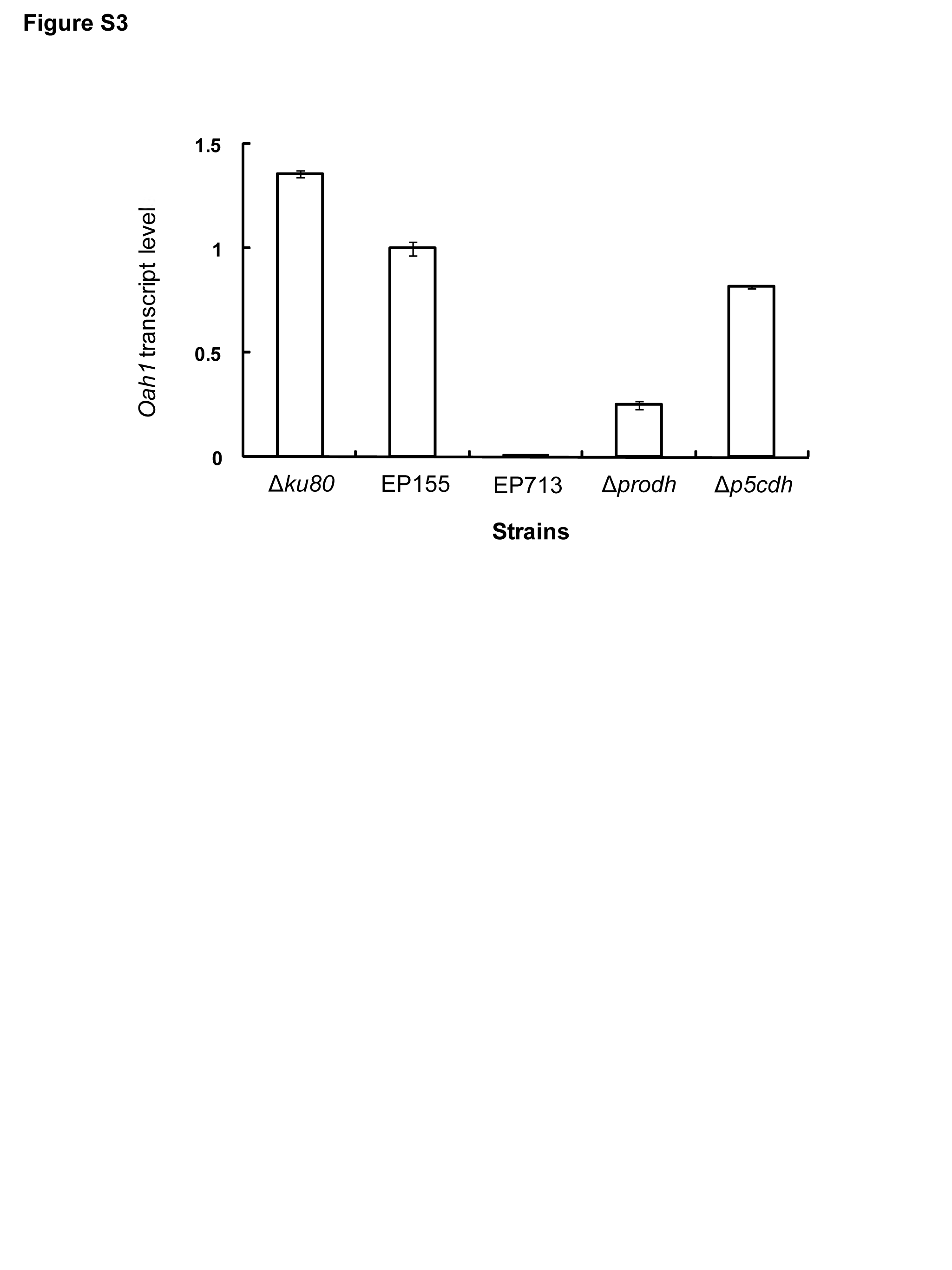

Supplement: Figure S3 — Quantification of the transcript level of Oah1. The strains were cultured on PDA at 25°C for 7 days, and mycelia were collected for mRNA isolation. The Oah1 transcript accumulation levels were determined by RT-PCR using the Oah1-specific primers oah1-Qf and oah1-Qr. The transcript level in EP155 was set at 1.0, and the corresponding levels in the other strains are expressed as a percentage of the levels in EP155. The values were calculated from three independent experiments. The error bars represent standard deviations. (TIF) [file pone.0073483.s003.tif]
