# Supplementary material for: Δ1-Pyrroline-5-Carboxylate/Glutamate Biogenesis Is Required for Fungal Virulence and Sporulation
Source: PLoS One. 2013 Sep 9;8(9):e73483. doi: 10.1371/journal.pone.0073483 (PMC3767830; doi:10.1371/journal.pone.0073483)
Supplement: Table S1 — Primers used in this work. (DOC) [file pone.0073483.s004.doc]

Table S1. Primers used in this work

| Gene name | Primer name | Sequence(5'→3') |
| --- | --- | --- |
| *Prodh* | prodh-f-*Eco*RⅠ | atagaattcatgagggccaaagcaccact |
|  | prodh-r-*Xba*Ⅰ | taatctagattacgccgtcacccccaatg |
|  | prodh-Δ21-f | gtgacaaccatctcttcatc |
|  | sdh-f-*Eco*RⅠ | gcggaattccaccacgagaataaagatgctatcgct |
|  | sdh-prodh-r | tgatgaagagatggttgtcactcgtgatctattatgtggt |
| *P5Cdh* | p5cdh-f-*Hind*Ⅲ | gcgaagcttatggcttctcgcagggtcagtctcc |
|  | p5cdh-r-*Xba*Ⅰ | gcctctagagacctcattgctcggatactcgac |
|  | p5cdh-Δ45-f | gtgctagcgaccttcaagac |
|  | sdh-f-*Hind*Ⅲ | gcgaagcttcaccacgagaataaagatgctatcgct |
|  | sdh-p5cdh-r | gtcttgaaggtcgctagcacctcgtgatctattatgtggt |
| *Put1* | put1-f-*Eco*RⅠ | atagaattccaccctagaaatgatagcttcc |
|  | put1-r-*Xba*Ⅰ | taatctagatcataggcctactctttttg |
| *Put2* | put2-f-*Hind*Ⅲ | gcgaagcttatgctatcagcaaggtgcctcaaat |
|  | put2-r-*Xba*Ⅰ | gcctctagattcataattcgatggatatttg |
| *Prodh* | prodh-A | tagccacggcattctgaggagcaac |
|  | prodh-B | ctcaaggtcaacaaatactacaagc |
|  | prodh-3 | tctttctagaggatccccgggtaccgttgactcgacggtggtatgc |
|  | prodh-2 | atatcatcttctgtcgacctgcaggctcgtgagacatgacagtacc |
|  | prodh-1 | tgaattgagcacagcatcctgc |
|  | prodh-4 | gtccgagatgctcatcgtcag |
|  | prodh-F | attgttcgatccaagcagcg |
| *P5Cdh* | p5cdh-A | atttagccgtcgtgacatcttcctc |
|  | p5cdh-B | ggtgtgagagagaaggtggtcgttc |
|  | p5cdh-3 | tctttctagaggatccccgggtaccggattggatggattgagctgt |
|  | p5cdh-2 | atatcatcttctgtcgacctgcaggctatccgagcaatgaggtctg |
|  | p5cdh-1 | gcatcccgtactaattcgct |
|  | p5cdh-4 | ccaaccgtctcctactactg |
|  | p5cdh-F | tgctcaccttgctgctcttggaagt |
| *Car1* | car1-A | ttgtcgtatagtatgtgctggcttg |
|  | car1-B | tcgactgggagggagaagcacctct |
|  | car1-1 | cgccaggaccctctttcgac |
|  | car1-4 | ttggtacgagaaggtggaag |
|  | car1-3 | tctttctagaggatccccgggtaccgtggtggtcgttaaagtggtt |
|  | car1-2 | atatcatcttctgtcgacctgcaggcgctgaataaaggaaagaga |
| *Car2* | car2-A | ccagagcccatgttgagaatgtgtt |
|  | car2-B | ctctcacgtaccatccgtcaagtac |
|  | car2-1 | ttcataagtcaaggagtgtt |
|  | car2-4 | gacactcgacctggagagtt |
|  | car2-3 | tctttctagaggatccccgggtaccggtacgagctggtcccaagat |
|  | car2-2 | atatcatcttctgtcgacctgcaggccacacaaagttgcacatgag |
| *Pro3* | pro3-A | cgcatttcagcctgaactcgtcatt |
|  | pro3-B | ggaacatgtaacggcctgtgtcgtt |
|  | pro3-1 | atcatagacaccgctgactt |
|  | pro3-4 | cgtgagttacttcccttctc |
|  | pro3-3 | tctttctagaggatccccgggtaccggtatgagacaggatgaatgt |
|  | pro3-2 | atatcatcttctgtcgacctgcaggctcgggtgatgagaggatgtg |
| *Pro2* | pro2-A | tggtccttgattgtgattcgtggtt |
|  | pro2-B | tcctccctctacccttacagacatc |
|  | pro2-1 | tgtgacggctcaacggtatg |
|  | pro2-4 | gtcgagttcgcaggcatcat |
|  | pro2-3 | tctttctagaggatccccgggtaccggtggtgcttgttgtggttgt |
|  | pro2-2 | atatcatcttctgtcgacctgcaggcaagtaagtaggggattcaag |
| *Pro1* | pro1-A | tccagcgaatatctagtgagccttg |
|  | pro1-B | cattatgtatgtcaccgacaagatg |
|  | pro1-1 | gcagatacagtagttctcgt |
|  | pro1-4 | tgacgagcgttgcgcaaact |
|  | pro1-3 | tctttctagaggatccccgggtaccgtggctcttcatgtctaccag |
|  | pro1-2 | atatcatcttctgtcgacctgcaggctcgccgagtatgagtccaag |
| *Put4* | put4-A | agtctagggagacccaccacatcat |
|  | put4-B | tgagagcatgatcgctggtgccatt |
|  | put4-1 | agccgataccttgtgagact |
|  | put4-4 | aggacgtggaggccgttgtc |
|  | put4-3 | tctttctagaggatccccgggtaccgatgtcggcgtaaccacttgc |
|  | put4-2 | atatcatcttctgtcgacctgcaggcaacgagacgttatgttgctc |
| *Put3* | put3-A | gtcaagatcctcggctgagtgtcat |
|  | put3-B | attctgataagcatctggcacgatc |
|  | put3-1 | agaaccaactgtgcactgtg |
|  | put3-4 | gatccgagacaacgcagatt |
|  | put3-3 | tctttctagaggatccccgggtaccgagtggagccaacaaggtatc |
|  | put3-2 | atatcatcttctgtcgacctgcaggcaagagcttgaatggccggtt |
| *Hph* | hph-f | cggtacccggggatcctctag |
|  | hph-r | gcctgcaggtcgacagaagatg |
| *18S* | CP18SF | acgctggcttcttagagggact |
|  | CP18SR | cagcacgacagagtttcacaag |
| *Prodh* | prodh-Qf | acggagacggaagtcgcatt |
|  | prodh-Qr | ctgcgaaggaggtacttcat |
| *P5Cdh* | P5Cdh-Qf | ttctacatcaactgcaagag |
|  | P5Cdh-Qr | actcttccttcatggtcctc |
| *Oah1* | oah1-Qf | cgatatgatcgccaacctcg |
|  | oah1-Qr | atccggacgaggtactcatc |
